# Discerning Endoscopic Severity of Inflammatory Bowel Disease by Scoping the Peripheral Blood Transcriptome

**DOI:** 10.1016/j.gastha.2024.02.009

**Published:** 2024-03-05

**Authors:** Jan Söderman, Sven Almer

**Affiliations:** 1 Department of Biomedical and Clinical Sciences, Linköping University, Linköping, Sweden; 2 Laboratory Medicine, Jönköping, Region Jönköping County, Sweden; 3 Department of Medicine, Solna, Karolinska Institutet, Stockholm, Sweden; 4 IBD-Unit, Division of Gastroenterology, Karolinska University Hospital, Stockholm, Sweden

**Keywords:** Crohn’s Disease, Gene Set Enrichment Analysis, RNA-seq, Ulcerative Colitis

## Abstract

**Background and Aims:**

Ulcerative colitis (UC) and Crohn’s disease (CD) are chronic inflammatory bowel diseases (IBDs) with an incompletely understood etiology and pathogenesis. Identification of suitable drug targets and assessment of disease severity are crucial for optimal management.

**Methods:**

Using RNA sequencing, we investigated differential gene expression in peripheral blood samples from IBD patients and non-inflamed controls, analyzed pathway enrichment, and identified genes whose expression correlated with endoscopic disease severity.

**Results:**

Neutrophil degranulation emerged as the most significant pathway across all IBD sample types. Signaling by interleukins was prominent in patients with active intestinal inflammation but also enriched in CD and UC patients without intestinal inflammation. Nevertheless, genes correlated to endoscopic disease severity implicated the primary cilium in CD patients and translation and focal adhesion in UC patients. Moreover, several of these genes were located in genome-wide associated loci linked to IBD, cholesterol levels, blood cell counts, and levels of markers assessing liver and kidney function. These genes also suggested connections to intestinal epithelial barrier dysfunction, contemporary IBD drug treatment, and new actionable drug targets. A large number of genes associated with endoscopic disease severity corresponded to noncoding RNAs.

**Conclusion:**

This study revealed biological pathways associated with IBD disease state and endoscopic disease severity, thus providing insights into the underlying mechanisms of IBD pathogenesis as well as identifying potential biomarkers and therapies. Peripheral blood might constitute a suitable noninvasive diagnostic sample type, in which gene expression profiles might serve as indicators of ongoing mucosal inflammation, and thus guide personalized treatment decisions.

## Introduction

Inflammatory bowel disease (IBD) comprises 2 heterogeneous subtypes, Crohn's disease (CD) and ulcerative colitis (UC), both characterized by recurrent gastrointestinal inflammation. IBD can be considered a disease spectrum rather than 2 distinct subtypes, with variations in gastrointestinal presentation, extraintestinal manifestations, disease progression, and treatment response.^1^, ^2^, ^3^ Although the etiology and pathogenesis of IBD remain unclear, it is widely accepted that a complex interplay of environmental, genetic, immunologic, and intestinal factors such as barrier properties and microbial community play a critical role.^4^ This complexity is evident from genome-wide association studies (GWASs) which have identified at least 241 risk loci: 48 for CD, 31 for UC, and 162 shared between the 2 subtypes.^5^ Transcriptomic studies have investigated dysregulated pathways in IBD using, for instance, peripheral blood,^6^^,^^7^ mucosal biopsies,^8^^,^^9^ and single cell samples of peripheral blood as well as intestinal mucosa.^10^ Dysregulated pathways and genetic susceptibilities offer insights into disease mechanisms as well as potential targets for IBD therapy. Common laboratory tests for IBD diagnosis (eg C-reactive protein, alkaline phosphatase, hemoglobin, platelet count, and calprotectin) lack specificity and are not suited for individualized treatments. Transcriptional signatures associated with disease progression and treatment response have been studied using peripheral blood^11^^,^^12^ and intestinal mucosa samples.^13^^,^^14^ While endoscopy is preferred for assessing mucosal inflammation and disease activity, it is invasive and associated with risks, whereas peripheral blood offers a more accessible, less invasive sample source. Despite IBD treatment advancements, a deeper understanding of IBD is needed to account for its heterogeneous nature and deliver personalized medicine. Here, we investigated gene expression changes in peripheral blood from CD and UC patients to decipher disease mechanisms, uncover possible therapeutic targets, and explore noninvasive indicators of mucosal inflammation and endoscopic disease severity in IBD.

## Material and Methods

### Study Samples

Peripheral blood specimens were collected using Tempus Blood RNA Tubes (Life Technologies, Carlsbad, California, USA) during routine endoscopy of adults investigated for confirmed CD (n = 17) or UC (n = 22) diagnosis or suspected gastrointestinal disorders (n = 28) (Table 1), and each patient’s transcriptional profile was examined using a single blood sample.

**Table 1:** Characteristics of Patients With Crohn's Disease (CD) and Ulcerative Colitis (UC), With or Without an Intestinal Inflammation (I and nI, Respectively), and Non-Inflamed Controls (Cntrl)^a^

Patient characteristics	CD.nI n = 5	CD.I n = 12	UC.nI n = 7	UC.I n = 15	Cntrl n = 28
Gender (male/female)	3/2	7/5	2/5	5/10	10/18
Age (y)^b^	21 (20–43)	47.5 (29–71)	46 (30–65)	35 (18–68)	37.5 (19–81)
Smoker (yes/previous/no/no data)	2/0/2/1	1/0/10/1	0/1/6/0	3/3/7/2	0/2/21/5
SES-CD^b^^c^	2 (0–2)	8 (1–18)	NA	NA	NA
UCEIS^b^^d^	NA	NA	1 (1–1)	5 (3–6)	0 (0–2)
SAES^b^^e^	NA	NA	4 (2–5)	14 (6–26)	0 (0–6)
Concomitant drug treatment^f^					
AP, TP	0	0	1	0	0
AS	1	1	4	2	0
AS, ATA	0	1	0	0	0
AS, CS	0	1	0	3	0
AS, CS, TP	0	0	0	2	0
AS, TP	1	0	0	0	0
ATA	0	1	0	0	0
ATA, CS, TP	0	0	0	1	0
ATA, TP	0	1	0	0	0
CS	1	3	0	1	0
CS, TP	0	0	0	1	0
TP	0	2	0	1	0
None	2	2	2	4	28

a Peripheral blood samples from CD and UC patients with either an actively inflamed intestinal mucosa (CD.I and UC.I) or a non-inflamed mucosa (CD.nI and UC.nI) were compared to samples obtained from a control group of patients (Cntrl) referred for endoscopic examination due to gastrointestinal symptoms (*eg* diarrhea, fecal blood, or abdominal pain; n = 22), anemia (n = 1) or screening for colorectal cancer (n = 5), with the following findings: diverticulosis (n = 3), polyps (n = 2), low-grade dysplasia adenomas (n = 1), colorectal cancer (n = 1), hemorrhoids (n = 1), or without any abnormal histopathological findings pathological findings (n = 20).

b Median (range) values are given.

c Simplified endoscopic activity score for CD patients. NA, not applicable.

d UC endoscopic index of severity. NA, not applicable.

e The sum of the segmental assessment of endoscopic severity regarding granularity, vascular pattern, ulceration, and bleeding-friability. NA, not applicable.

f The following drugs were used individually or in combination: allopurinol (AP; 1 UC.nI), aminosalicylates (AS; 2 CD.nI, 3 CD.I, 4 UC.nI and 7 UC.I), anti-TNF-antibodies (ATA; 3 CD.I, 1 UC.I), corticosteroids (CS; 1 CD.nI, 4 CD.I, and 8 UC.I), thiopurines (TP; 1 CD.nI, 3 CD.I, 1 UC.nI and 5 UC.I).

CD and UC patients were deemed actively inflamed (CD.I and UC.I) based on biopsy-confirmed inflammation by an experienced endoscopist (S.A.). Those without signs of inflammation in all biopsies were termed non-inflamed (CD.nI and UC.nI). Disease severity was assessed using the simplified endoscopic activity score (SES-CD) for CD patients,^15^ and for UC the well-established UC endoscopic index of severity (UCEIS),^16^ and further using the sum of the segmental assessment of endoscopic severity (SAES) regarding granularity, vascular pattern, ulceration, and bleeding friability.^17^

The non-inflamed control group (Cntrl) underwent endoscopy because of gastrointestinal symptoms (n = 22), anemia (n = 1), or colorectal cancer screening due to acromegaly (n = 5). Eight controls had findings such as diverticulosis (n = 3), polyps (n = 2), or others. The remaining 20 controls had no abnormalities.

The study received approval from the Linköping Regional Ethical Review Board (Dnr 2011/201-31, amendment Dnr 2013/211-32). All participants gave written informed consent.

### Sample Preparation and RNA Sequencing

Peripheral blood samples were processed using the Tempus Spin RNA isolation kit (Life Technologies) according to the manufacturer’s instructions. RNA samples were assessed and sequenced as previously described,^9^ except with sequencing libraries constructed using TruSeq Stranded total RNA with Ribo-Zero Globin (Illumina, San Diego, CA, USA).

### Statistical Analysis and Bioinformatics

Unless specified, data processing and analysis utilized R packages for R 4.2.0 (released April 22, 2022, CRAN: https://cran.r-project.org/). Packages were acquired from Bioconductor (https://bioconductor.org/) or CRAN, with specific names and versions detailed in subsequent method sections. *P* values were adjusted for multiple testing using the Benjamini–Hochberg method to control the false discovery rate. Adjusted *P* values < .05 were considered significant.

### Analysis of Differential Expression Using RNA-Seq Data

Reads were aligned to the GRCh38 assembly from Ensembl (http://ftp.ensembl.org/pub/release-106/fasta/homo_sapiens/dna/; accessed June 22, 2022) and mapped to 61,552 Ensembl gene IDs (http://ftp.ensembl.org/pub/release-106/gtf/homo_sapiens/) using Rsubread v2.10.2. The median number of mapped reads was 22.8 million (5%–95% range: 14.4–29.0 million reads).

RNA-seq count data were analyzed for differentially expressed genes (DEG) as previously described^9^ using DESeq2 v1.36.0. Only genes with full annotations (sourced via AnnotationDbi v1.58.0 and org.Hs.eg.db v3.15.0) and passing a low expression filter were included, yielding 24,153 genes. These were tested against a log2-fold-change threshold of zero. Group differences were examined by combining variables of interest, such as disease and inflammation status, while adjusting for gender.

### Proportions of Peripheral Blood Cell Types

Peripheral blood cell types were estimated using gene expression deconvolution, leveraging the immunoStates basis matrix^18^ and MetaIntegrator v2.1.5 (courtesy of Dr Aditya Rao, Stanford University). Of the expressed genes, 310 from 317 immunoStates matrix genes were available for deconvolution. Before deconvolution, count data were normalized using the transcript per million method and then log2 transformed. Cell types absent in over half the samples in the analyzed groups were omitted from statistical analysis. Cell type proportions were compared using the Wilcoxon rank sum test, with *P* values adjusted for multiple tests.

### Gene Set Analysis

When comparing IBD patients and controls, ranked gene lists were created by multiplying the sign of the fold change with the absolute log of the unadjusted *P* value. These ranked lists, with upregulated genes at the top and downregulated at the bottom, were based on all 24,153 genes analyzed for differential expression.

Gene set enrichment analysis of Reactome pathways (https://reactome.org/) was performed using the ranked lists and the compareCluster and gsePathway functions (1 × 10^6^ permutations, gene set size 5–500) from clusterProfiler v4.4.3 and ReactomePA v1.40.0.

Genes associated to endoscopic grading underwent over-representation analysis (ORA) using enrichGO and enrichPathway functions from clusterProfiler and ReactomePA packages, respectively. enrichGO used predefined gene sets from the Gene Ontology domains (cellular component, biological process, and molecular function: http://geneontology.org/), while enrichPathway used predefined Reactome gene sets.

### Network Analysis and Visualization

Pathways were clustered using the Cytoscape software v3.9.1 with the plugins EnrichmentMap v3.3.4 and clusterMaker2 v2.2 employing the Markov clustering algorithm. For full network connectivity, clustering was based on the original gene sets of identified pathways, not just the subset of genes that contributed to the enrichment, that is, the core enriched genes (CEG) of a given pathway.^19^ To standardize cluster naming, pathways (identified at an adjusted *P* < .05) from all group comparisons were integrated into one network. The name of each cluster was taken from the pathway with the lowest adjusted *P* value.

A clustered dotplot was devised by merging pathways identified using compareCluster (adjusted *P* < .005 to reduce the number of visualized pathway clusters) with cluster data from the Cytoscape analysis (adjusted *P* < .05). Within each pathway cluster, the median percentage of CEG contributing to the enrichment score and the median normalized enrichment score were determined. These values were represented by dot size and color, respectively. For clarity, the dotplot underwent hierarchical clustering (Euclidean distance matrix) based on the median normalized enrichment score values and was displayed as a dendrogram. Before clustering, missing values were set to zero. The dotplot was produced using the R packages stats v4.2.0, ggplot2 v3.3.6, and cowplot v1.1.1.

### Cytokines and Cytokine Receptors

A combined list of 312 genes encoding cytokines and cytokine receptors was derived from the Cytokine Registry File (https://www.immport.org/resources/cytokineRegistry; last updated November 2015, accessed March 21, 2023) and from reviewed human protein entries in the cytokine category from UniProtKB (https://www.uniprot.org/keywords/KW-0202; accessed March 21, 2023), and DEG and CEG were cross-referenced against this list.

### Endoscopic Disease Severity and Gene Expression in Peripheral Blood

Expression of genes differentially expressed in CD.I and/or UC.I were examined for correlations with endoscopic grading scores in both inflamed and non-inflamed patient samples. Kendall's tau correlation coefficient (stats v4.2.0: cor.test function) was used for statistical association. The genes that were given priority, based on a low *P* value for the correlation or ORA results, were further evaluated for genetic associations using the catalog of human GWASs (https://www.ebi.ac.uk/gwas; accessed on May 26, 2023). Additional gene information was sourced from NCBI Gene (https://www.ncbi.nlm.nih.gov/gene) and UniProtKB (https://www.uniprot.org/), both accessed on May 26, 2023. In the GWAS Catalog, associations with a *P* < .00000005 were deemed significant, which is a commonly accepted threshold based on a Bonferroni correction for the number of independent common variants across the genome.^20^

Correlations for CD patients were based on SES-CD and CD.I DEG. For UC, correlations involved UC.I DEG and either SAES or UCEIS. In order to assess the overall endoscopic severity in both CD and UC patients and the DEG common to both conditions, individual scores (SES-CD, SAES, and UCEIS) were rescaled to a range of 1–20 using scales v1.2.1 and the rescale function. Unified, rescaled IBD scores were created by merging the rescaled SES-CD with either the rescaled SAES or the rescaled UCEIS.

## Results

### Peripheral Blood Cell Populations

Deconvolution revealed no significant differences in the proportions of blood cell types for non-inflamed patients with CD or UC compared to controls (Table A1), whereas patients with an active inflammation had reduced proportions in the total compartments of B cells, T cells, and natural killer cells and an increased proportion of the monocyte compartment (range of adjusted *P* < .001–0.041). In the granulocyte compartment, both CD.I and UC.I patients displayed an increased proportion of basophils (adjusted *P* = .028 and < 0.001, respectively), and UC.I an increased proportion of neutrophils (adjusted *P* = .023), with a borderline significance for in CD.I (adjusted *P* = .051).

### Differentially Expressed Genes

In non-inflamed CD and UC patients, one and 2 genes, respectively, were downregulated (Table A2), whereas inflamed CD patients displayed 2033 DEG (Table A2; 959 and 1074 upregulated and downregulated, respectively) and inflamed UC patients displayed 3854 genes (Table A2; 2504 and 1350 upregulated and downregulated, respectively). Among DEG, 915 genes were shared by CD.I patients (646 and 269 upregulated and downregulated, respectively) and UC.I patients (647 and 268 upregulated and downregulated, respectively), and all but one gene showed concordant regulation.

### Gene Set Analysis

**Figure 1:**
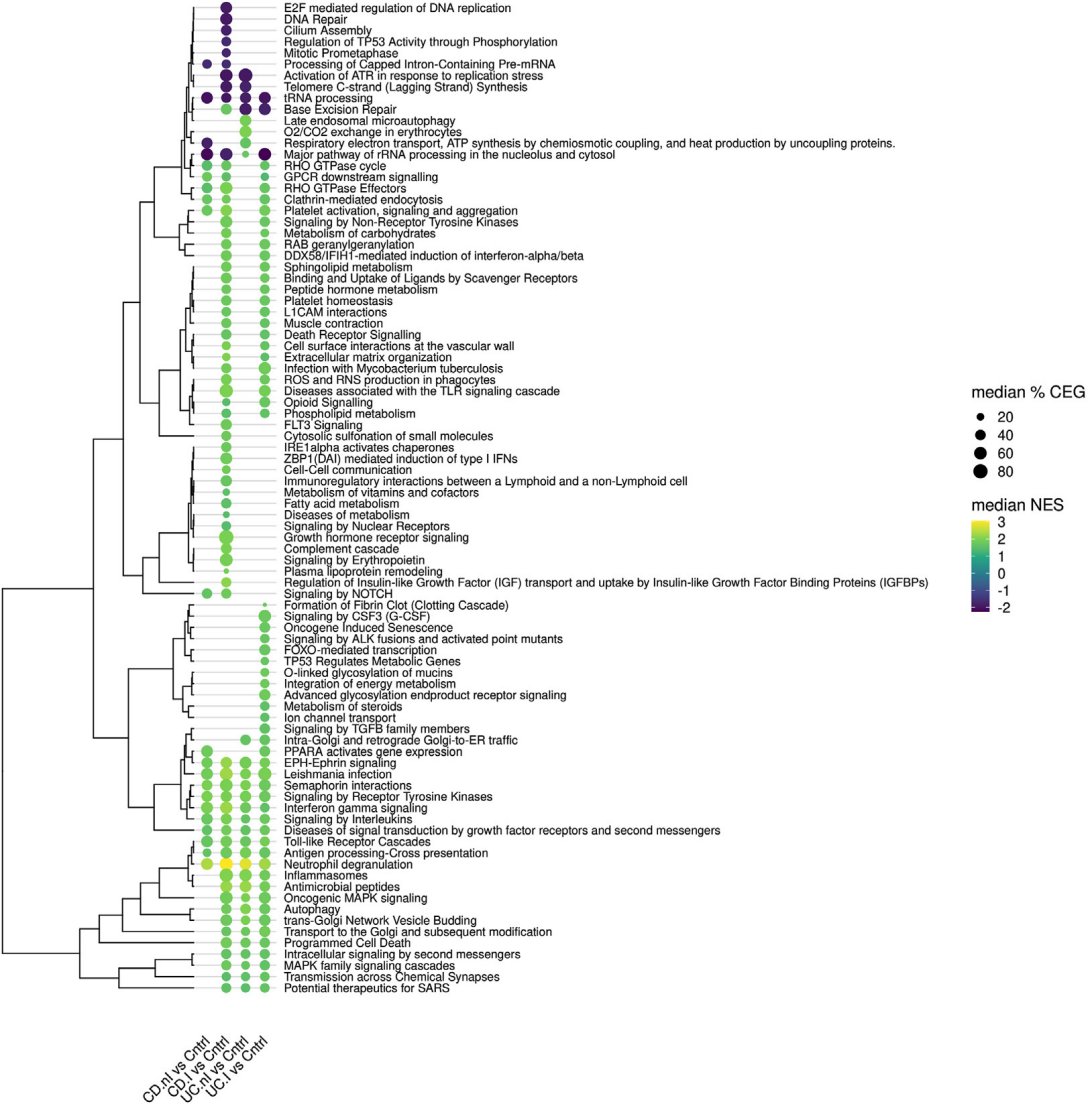
Gene set enrichment based on gene expression in peripheral blood samples from patients with ulcerative colitis (UC) or Crohn's disease (CD), with (CD.I, n = 12; UC.I, n = 15) or without (CD.nI, n = 5; UC.nI, n = 7) active inflammation, were compared to controls without intestinal inflammation (Cntrl, n = 28). The most significant (adjusted *P* < .005) gene set enrichment results are visualized as a dot plot, with Reactome pathway clusters along the y-axis and pairwise group comparisons along the x-axis. For each pathway cluster, the median value of the proportions of core enriched genes (CEG) was mapped to the dot size, and the median normalized enrichment score (NES) was mapped to the dot color, where a positive or a negative score corresponds to pathway clusters with upregulated or downregulated genes, respectively. Pathway clusters were further arranged (dendrogram) based on similarities in their median NES values.

Using peripheral blood samples from CD.nI compared to controls, gene set enrichment analysis (adjusted *P* < .05) identified a total of 160 pathways, distributed among 40 clusters and 6 singletons (Table A3). The corresponding numbers for CD.I, UC.nI, and UC.I were 542 (86 clusters and 14 singletons), 363 (62 clusters and 4 singletons), and 541 (80 clusters and 17 singletons) pathways. Prior to visualization, the complexity of the data was reduced by only including pathways with an adjusted *P* < .005, and by summarizing pathway clusters by their median values. The more stringent *P* value cutoff resulted in 69 pathways for CD.nI (31 upregulated and 38 downregulated), and 275 (226/49) for CD.I, 108 (101/7) for UC.nI, and 240 (195/45) for UC.I (Table A3), which collectively represented 88 pathway clusters and singletons that showed both differences and similarities in pathway expression across the 4 group comparisons (Figure 1).

#### Biological pathway clusters enriched in all 4 sample types

Among the 88 pathway clusters and singletons, twelve were enriched in all 4 blood IBD sample types, 10 were concordantly upregulated, “tRNA processing” was concordantly downregulated, and “major pathway of rRNA processing in the nucleolus and cytosol” was upregulated in samples from UC.nI but downregulated in the other IBD samples (Figure 1, Table A3).

Neutrophil degranulation, which was found among the 10 concordantly upregulated pathways, was the most significant of all pathways identified in all IBD sample types (Table A3). Otherwise, the most significant CD.I pathway clusters were “signaling by interleukins”, “Leishmania infection”, and “signaling by receptor tyrosine kinases,” whereas top UC.I pathway clusters were “major pathway of rRNA processing in the nucleolus and cytosol,” “signaling by interleukins,” and “diseases of signal transduction by growth factor receptors and second messengers.” For samples from non-inflamed IBD patients, the most significant pathway cluster was for CD.nI “major pathway of rRNA processing in the nucleolus and cytosol” and for UC.nI “antigen processing-cross presentation.” The remaining pathway clusters were “Toll-like receptor cascades,” ”interferon gamma signaling”, ”EPH-ephrin signaling,” and “semaphorin interactions.”

Although these pathway clusters were found among all 4 IBD groups, certain clusters showed large differences in the number of enriched pathways for different sample types, for example, the pathway clusters “antigen processing-cross presentation” and “major pathway of rRNA processing in the nucleolus and cytosol” (Table A3).

#### Upregulated biological pathway clusters common to samples from inflamed CD and UC patients

In addition to the pathway clusters enriched in all 4 sample types, samples from CD.I and UC.I patients showed upregulation for another 34 clusters, and 5 and 11 of these were also upregulated in samples from CD.nI and UC.nI patients, respectively (Figure 1, Table A3). No additional clusters were downregulated in samples from both CD.I and UC.I patients.

Among these pathway clusters, “platelet activation, signaling, and aggregation” was the most significant process of both CD.I and UC.I. Otherwise, the most significant enrichments in CD.I were “extracellular matrix organization” and “antimicrobial peptides,” whereas UC.I was more strongly associated with “transport to the Golgi and subsequent modification,” “RHO GTPase cycle,” and “diseases associated with the TLR signaling cascade.” Also, both sample types also showed strong enrichment, for example, “programmed cell death,” “metabolism of carbohydrates,” and “MAPK family signaling cascades.”

#### Biological pathway clusters enriched in the peripheral blood of patients with CD displaying an inflamed bowel but not observed in those with UC

Sixteen upregulated and 8 downregulated pathway clusters (or singletons) were enriched in blood samples from inflamed CD patients but not in samples from inflamed UC patients (Figure 1, Table A3).

The most significant upregulated pathway clusters concerned regulation of insulin-like growth factors by binding proteins and NOTCH signaling. Additional upregulated pathways encompassed, for example, cell-cell communication, the complement system, signaling by erythropoietin and FLT3, and metabolism of fatty acids.

The most significant downregulated pathway cluster was “Cilium Assembly.” Otherwise, the majority of downregulated pathway cluster were involved in DNA repair, telomere synthesis, the mitotic prometaphase, and replication stress.

#### Biological pathway clusters enriched in the peripheral blood of patients with UC displaying an inflamed bowel but not observed in those with CD

Fourteen upregulated and one downregulated pathway clusters (or singletons) were enriched in peripheral blood samples from inflamed UC patients but not in samples from inflamed CD patients (Figure 1, Table A3). The most significant pathway clusters were the upregulated “intra-Golgi and retrograde Golgi-to-ER traffic” and the downregulated “base excision repair.” Additional upregulated pathway clusters included, for example, “PPARA activates gene expression” (ie metabolism of fatty acids and lipids), “integration of energy metabolism,” and “O-linked glycosylation of mucins.”

On a side note, the pathway cluster “base excision repair” (Figure 1) appeared as upregulated in CD.I samples. However, these upregulated CD.I pathways did not concern base-excision repair but merely co-clustered due to the presence of histone H3-related processes.

#### Biological pathway clusters enriched in samples from non-inflamed CD and UC patients

Samples from CD.nI patients were enriched for 21 pathway clusters (17 upregulated and 4 downregulated), and UC.nI were enriched for 30 pathway clusters (26 upregulated and 4 downregulated) (Figure 1, Table A3).

The most significant pathway cluster of CD.nI was an upregulation of “neutrophil degranulation” but also the downregulated “major pathway of rRNA processing in the nucleolus and cytosol.” Additional highly significant clusters included, for example, downregulated “tRNA processing” as well as upregulated “platelet activation, signaling, and aggregation” and “Leishmania infection.”

The most significant pathway cluster of UC.nI was the upregulated “neutrophil degranulation” but also the upregulated “antigen processing-cross presentation,” “autophagy,” and “antimicrobial peptides.” The most significant downregulated pathway clusters were “tRNA processing,” “telomere C-strand (lagging strand) synthesis,“ and “activation of ATR in response to replication stress.”

One pathway cluster was uniquely enriched in blood samples from both CD.nI and UC.nI patients. This pathway cluster concerned mitochondrial respiratory electron transport and ATP synthesis and was downregulated in CD.nI samples but upregulated in UC.nI samples.

### Neutrophil Degranulation

**Figure 2:**
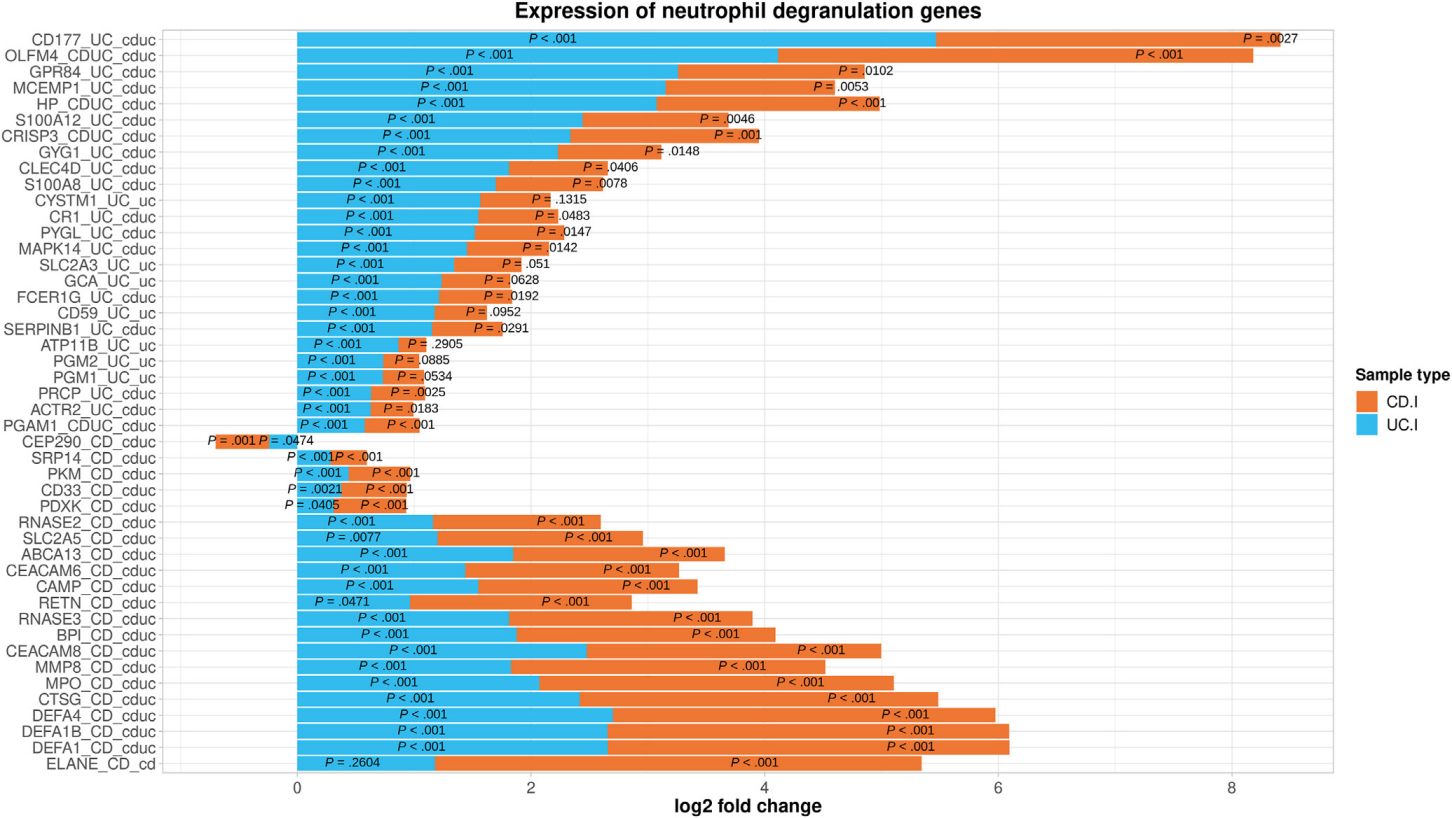
Expression profiles of the 25 most significant differentially expressed neutrophil degranulation-associated genes in peripheral blood from Crohn's disease (CD) and ulcerative colitis (UC) patients with intestinal inflammation (CD.I, n = 12; UC.I, n = 15). For each gene, the expression in CD and UC samples is described in relation to its expression in peripheral blood samples from controls without intestinal inflammation (Cntrl, n = 28) and is expressed as a symmetric log^2^-fold change (x-axis), where a positive value indicates an increased expression, while a negative value indicates a decreased expression. Adjusted *P* values for differential expression (compared to control samples) are indicated within the bars for each of the 2 sample types. Each gene (y-axis) has a suffix indicating whether the gene was among the top 25 DEG in samples from CD.I, UC.I, or both (indicated by capital letters CD, UC, or CDUC), and whether or not each gene was DE in CD.I, UC.I, or both (indicated by lowercase letters cd, uc, or cduc).

Of 482 genes associated with the pathway “neutrophil degranulation”, 249, 308, 245, and 238 genes were present among CEGs identified in peripheral blood samples, respectively, from CD.nI, CD.I, UC.nI, and UC.I (Table A3). Furthermore, 157 and 243 of the 482 genes were present among CD.I and UC.I DEG, respectively. The most significant DEG in CD.I patients were MPO, OLFM4, DEFA1, DEFA1B, and MMP8, while in UC.I patients GYG1, MCEMP1, CD177, S100A12, and HP were most strongly associated (Figure 2). ELANE, OLFM4, and 3 defensin genes (DEFA1, DEFA1B, and DEFA4) displayed the largest fold change differences in CD.I compared to samples from control patients, whereas in UC.I the genes with the largest fold change difference were CD177, OLFM4, GPR84, MCEMP1, and HP. In the combined set of top 25 DEG (in total 46 genes) from CD.I and UC.I (Figure 2), ELANE was the only gene that showed no differential expression in UC.I, while 7 of the genes showed no differential expression in CD.I. CEP290 was the only downregulated DEG in this set of genes and was present among the top 25 CD.I DEG.

### Cytokine and Cytokine Receptor Genes

**Figure 3:**
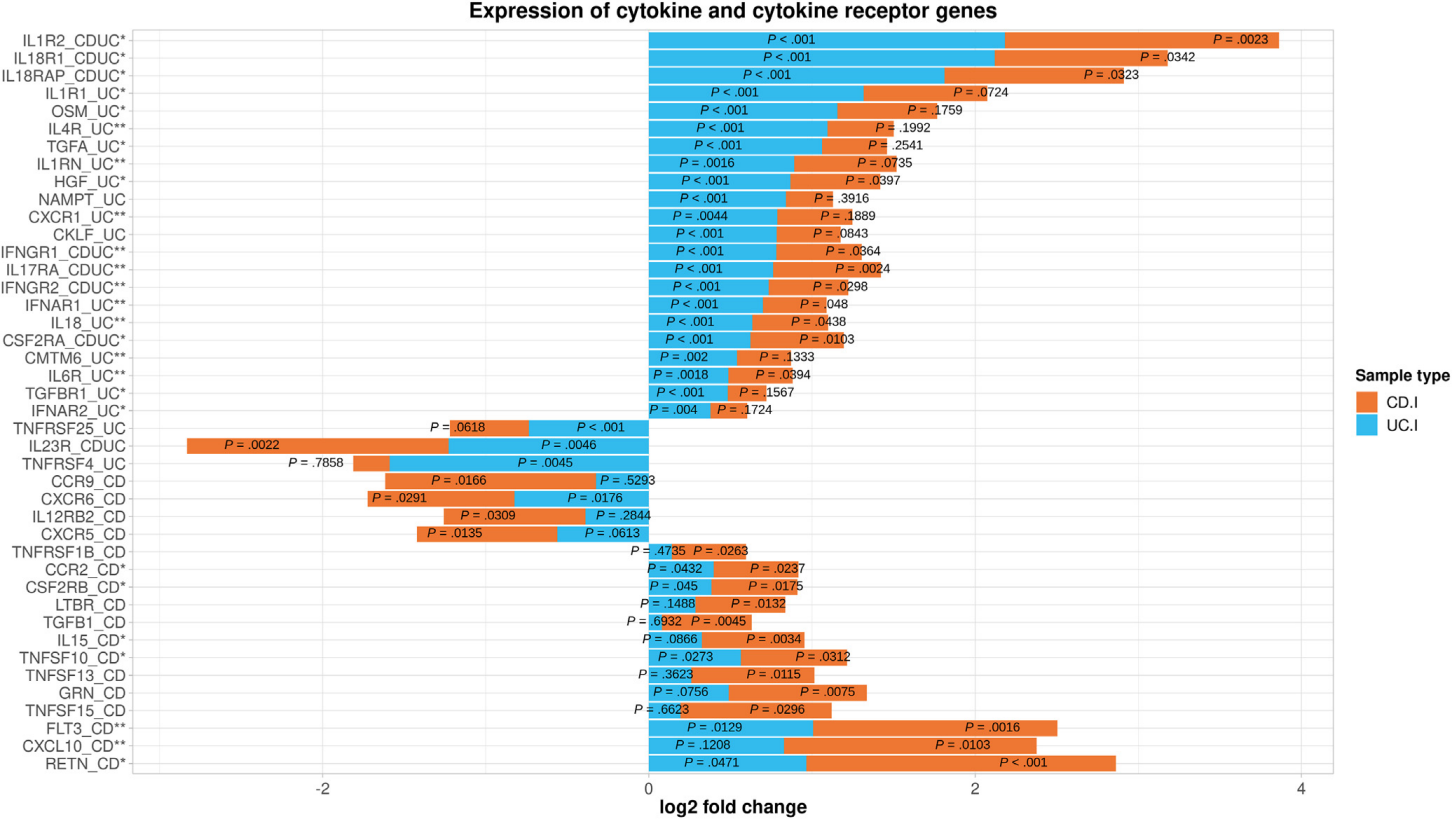
Expression profiles of the 25 most significant differentially expressed cytokine and cytokine receptor genes in peripheral blood from Crohn's disease (CD) and ulcerative colitis (UC) patients with an intestinal inflammation (CD.I, n = 12; UC.I, n = 15). For each gene, the expression in CD and UC samples is described in relation to its expression in peripheral blood samples from controls without intestinal inflammation (Cntrl, n = 28) and is expressed as a symmetric log^2^-fold change (x axis), where a positive value indicates an increased expression, while a negative value indicates a decreased expression. Adjusted *P* values for differential expression (compared to control samples) are indicated within the bars for each of the 2 sample types. Each gene (y-axis) has a suffix indicating whether the gene was among the top 25 cytokine and cytokine receptor genes in samples from CD.I, UC.I, or both. Additionally, a single asterisk indicates that the gene was found among core enrichment genes common to both CD.I and UC.I, while a double asterisk indicates that the gene was also found among CEG of all 4 inflammatory bowel disease sample types.

**Figure A1:**
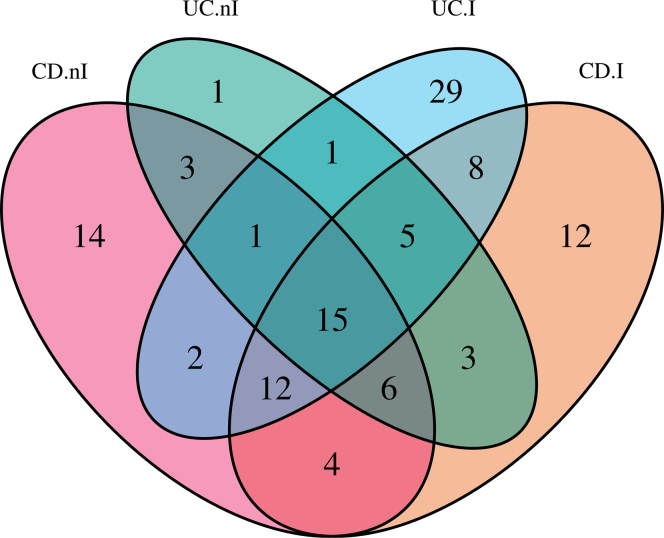
Commonalities and differences in core enriched genes encoding cytokines and cytokine receptors in peripheral blood samples from Crohn's disease (CD) and ulcerative colitis (UC) patients assessed as non-inflamed (CD.nI and UC.nI) or actively inflamed (CD.I and UC.I).

Within the pathway cluster “signaling by interleukins,” 39 cytokine and cytokine receptor genes were detected as part of the CEG (Table A4; CEG with an asterisk reference mark), and 6 of these were identified in all 4 group comparisons (ie, IL1B, IL1RN, and IL18 of the interleukin-1 family, and IL4R, IL6R, and CXCL10). Beyond this, cytokine CEGs were most prevalent in “MAPK family signaling cascades” (13, 9 and 7 genes in UC.I, CD.I, and UC.nI, respectively), “antigen processing-cross presentation” (eg12 genes in CD.I and 9 in UC.I), “GPCR downstream signaling” (eg 11 genes in CD.nI and 9 in CD.I), with additional contribution from DEG. Overall, 116 unique cytokine and cytokine receptor genes with partially overlapping expression profiles were identified for the different sample types (eg 40 genes were in common between CD.I and UC.I; Figure A1). The most significant differentially expressed cytokine and cytokine receptor genes in CD.I patients were RETN, FLT3, IL23R, IL1R2, and IL17RA (all but IL23R were upregulated), while UC.I showed the strongest association with an upregulation of IL18R1, IL1R2, IL4R, IFNAR1, and IL18RAP (Figure 3). Among the top 25 cytokine and cytokine receptor genes for CD.I samples, 2 (FLT3 and CXCL10) were discovered within the CEG of all 4 IBD sample types. For UC.I, 7 of the top 25 genes were found among CEG of all sample types (ie IL4R, IFNAR1, IL18, IL1RN, IL6R, CMTM6, and CXCR1). Three genes that were present in both lists (IL17RA, IFNGR2, and IFNGR1) were found among CEGs of all sample types.

### Endoscopic Disease Severity and Gene Expression

**Figure A2:**
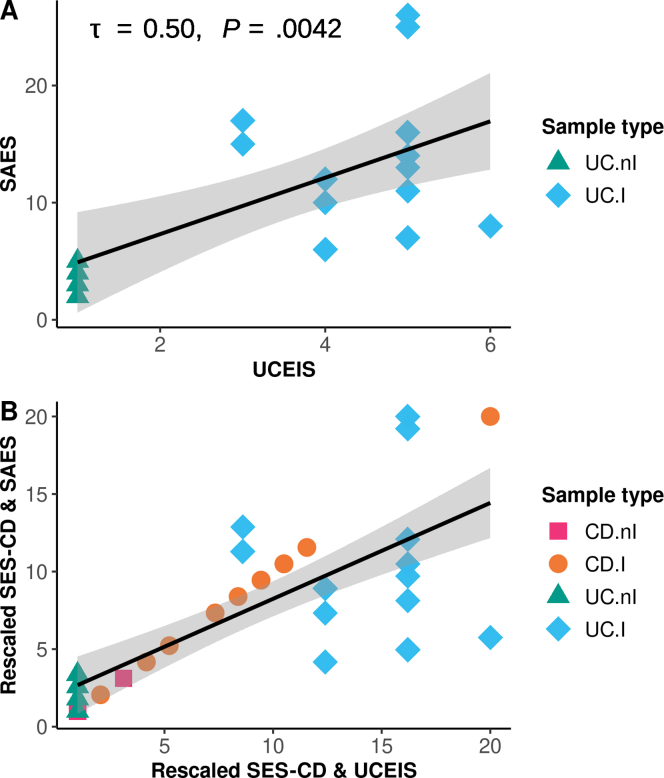
Degree of association between different endoscopic disease severity scales. (A) Correlation between SAES and UCEIS for UC patients (UC.nI, n = 6; UC.I, n = 15) measured using Kendall's tau correlation coefficient. (B) Visualization of the relationship between rescaled IBD scores (ie rescaled SES-CD combined with rescaled UCEIS, or rescaled SES-CD combined with rescaled SAES) for CD and UC patients (CD.nI, n = 5; CD.I, n = 12; UC.nI, n = 6; UC.I, n = 15).

In UC, disease severity was graded using both the UCEIS scale and the SAES scale, with a moderate agreement between the 2 (Kendall's tau = 0.50, *P* = .0042; Figure A2A). For CD patients, disease severity was graded using the SES-CD scale. Genes differentially expressed in peripheral blood samples from non-inflamed controls and CD or UC patients with intestinal inflammation were investigated for correlations between gene expression levels and endoscopic grading. Endoscopic findings were rescaled to a common range (Figure A2B), to explore the relationship between disease severity of CD and UC patients and DEG shared by CD and UC patients with active intestinal inflammation.

**Figure 4:**
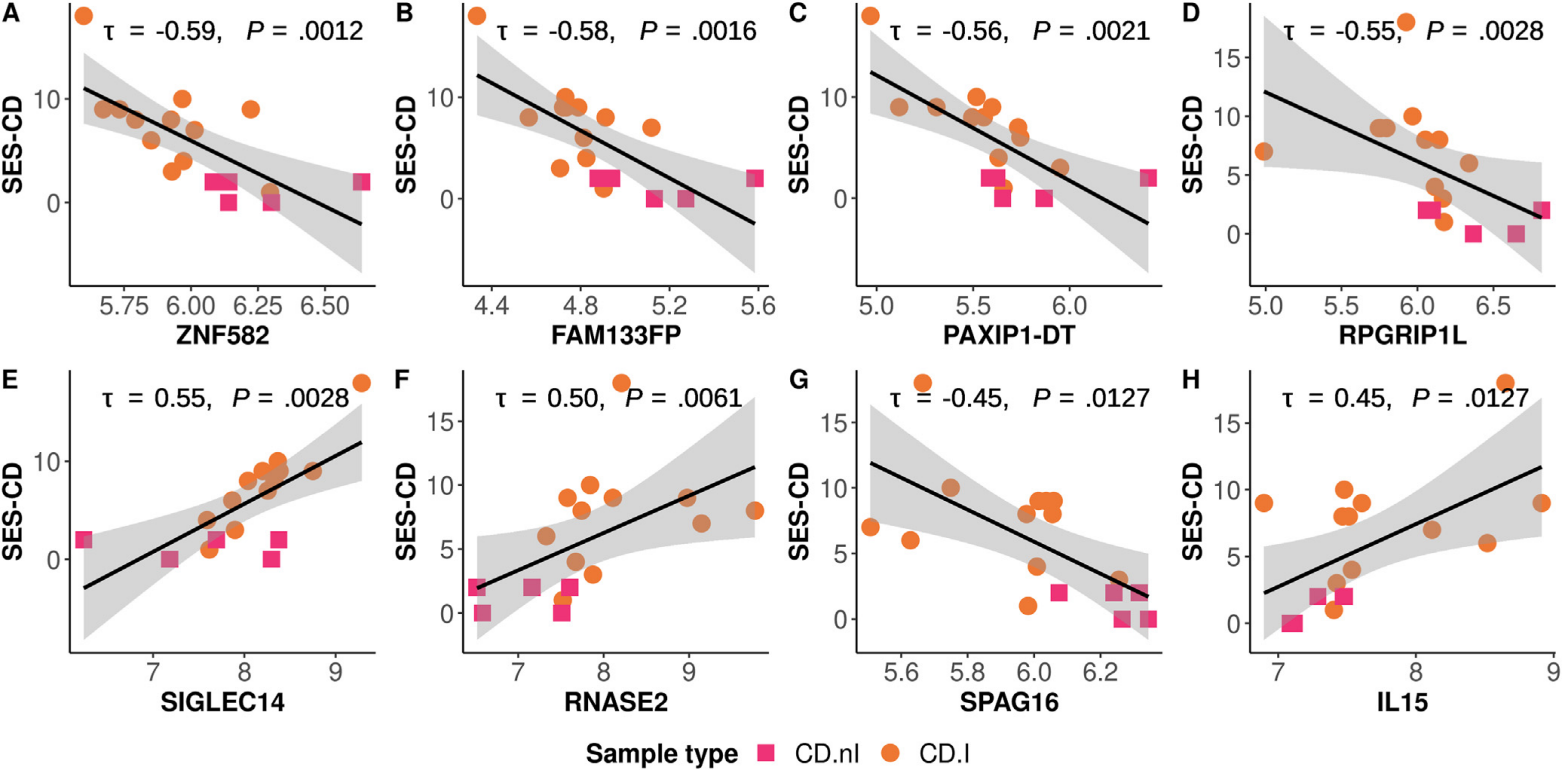
Degree of association between peripheral blood gene expression and endoscopic disease severity for CD patients (SES-CD; CD.nI, n = 5; CD.I, n = 12), as measured using Kendall's tau correlation coefficient. The plots are arranged in descending order of significance (A–H) based on the correlation coefficient.

Regarding SES-CD, none of the correlations to CD.I DEG passed adjustment for multiple testing, albeit 80 genes showed a nominal *P* < .05 (Table A5). The 3 most significant correlations involved a coding gene ZNF582 and 2 noncoding genes FAM133FP and PAXIP1-DT and exhibited a negative association with the endoscopic grading (Figure 4). The 80 genes included one cytokine gene (IL15) and 6 that belonged to the “neutrophil degranulation” pathway, of which SIGLEC14 and RNASE2 showed the strongest correlation (Figure 4). ORA identified the ciliary transition zone as a significant cellular component (Table A6), for example, RPGRIP1L and SPAG16 (Figure 4).

**Figure 5:**
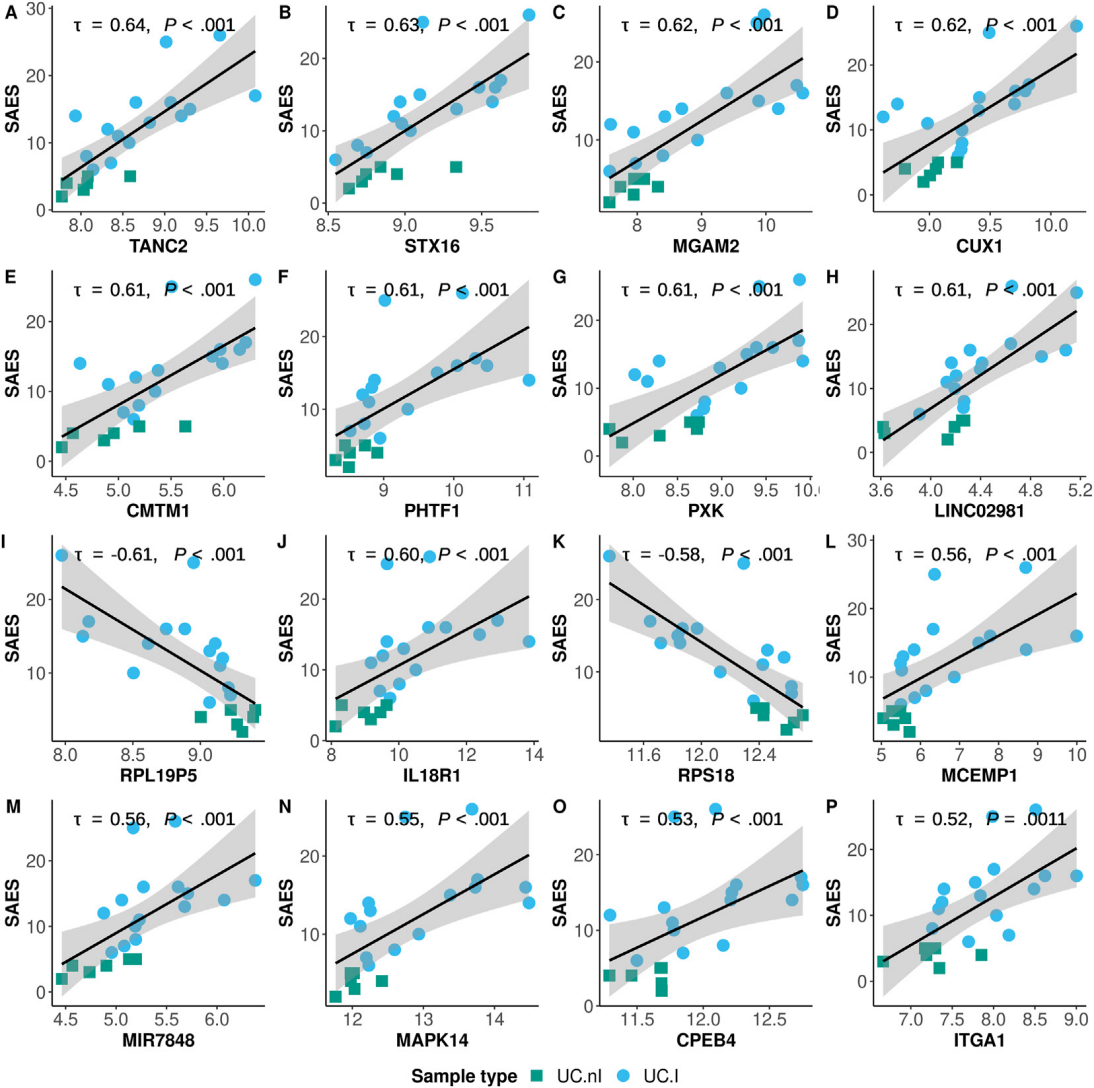
Degree of association between peripheral blood gene expression and endoscopic disease severity for UC patients (SAES; UC.nI, n = 6; UC.I, n = 15), as measured using Kendall's tau correlation coefficient. The plots are arranged in descending order of significance (A–P) based on the correlation coefficient.

With respect to UC patients and UC.I DEG, the SAES scale rendered 422 correlations that passed adjustment for multiple testing (Table A5), compared to the UCEIS scale that resulted in 253 correlations with a nominal *P* < .05 (Table A5). There was an overlap of 89 genes between the 2 sets. Among the 422 genes identified using the SAES scale, there were 4 positively correlated cytokine and cytokine receptor genes (eg CMTM1 and IL18R1), and 22 neutrophil degranulation-related genes (21 positively correlated, eg, MCEMP1 and MAPK14) (Figure 5). ORA identified significant processes and cellular components involved in cytoplasmic translation (eg RPS18 and CPEB4), focal adhesion (eg STX16 and ITGA1), and neutrophil granules and neutrophil degranulation (Table A6, Figure 5). Additional high-ranking coding genes included, for example, TANC2, MGAM2, CUX1, PHTF1, and PXK, and noncoding genes such as long intergenic nonprotein coding RNAs (eg LINC02981), pseudogenes (eg RPL19P5) and microRNAs (eg MIR7848) (Figure 5).

**Figure 6:**
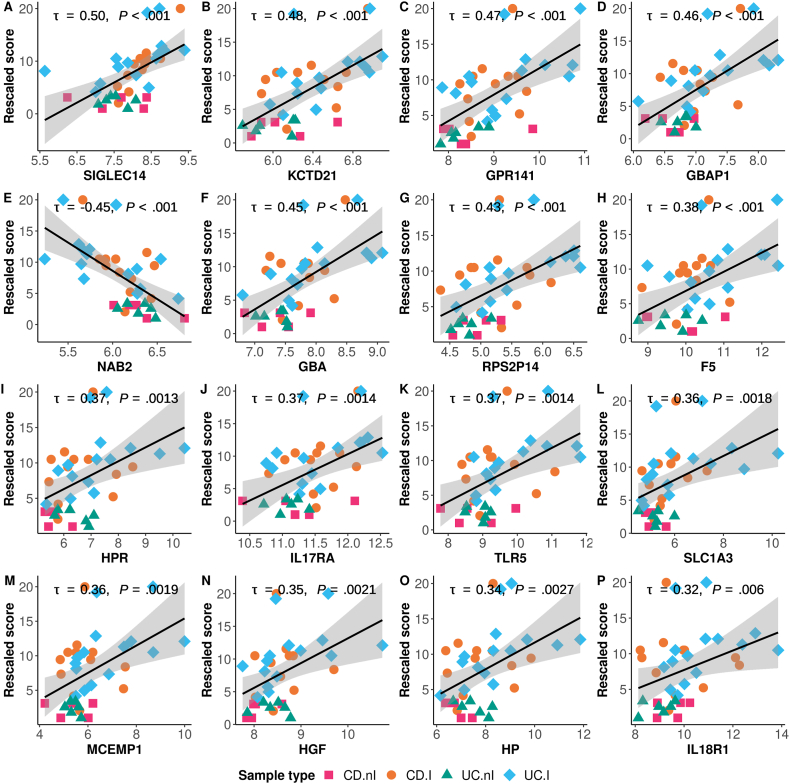
Degree of association between peripheral blood gene expression and a rescaled IBD endoscopic disease severity score based on the SES-CD scale for CD patients (CD.nI, n = 5; CD.I, n = 12) and the SAES scale for UC patients (UC.nI, n = 6; UC.I, n = 15), as measured using Kendall's tau correlation coefficient. The plots are arranged in descending order of significance (A–P) based on the correlation coefficient.

Using a rescaled IBD score based on the SES-CD scale for CD patients and the SAES scale for UC patients, and DEG shared between CD.I and UC.I patients, resulted in 119 correlations that passed adjustment for multiple testing (Table A5), whereas the version based on the UCEIS scale resulted in 99 correlations with a nominal *P* < .05 (Table A5). Among the 119 genes identified using the rescaled IBD score, there were 3 positively correlated cytokine and cytokine receptor genes (ie IL17RA, HGF, and IL18R1) and 21 positively correlated neutrophil degranulation-related genes (eg SIGLEC14, MCEMP1, and HP) (Figure 6). ORA identified significant processes and cellular components related to neutrophil granules, and possibly to platelet granules (eg F5) (Table A6, Figure 6). Additional high-ranking coding genes included, for example, KCTD21, GPR141, NAB2, GBA, HPR, TLR5, and SLC1A3, and pseudogenes (eg GBAP1 and RPS2P14) (Figure 6).

### GWAS Significant Traits and Genes Correlated With Endoscopic Disease Severity

Genes with expression levels that correlated with endoscopic grading scores were analyzed for their association with traits found in human GWASs.

According to the GWAS Catalog, a number of the genes that correlated with endoscopic disease severity were present in GWAS risk loci for CD, UC, or both disease subtypes. Regarding the SAES scale, PHTF1, IL18R1, and CPEB4 resided within IBD risk loci. For the rescaled IBD score, the pseudogene GBAP1 was in a risk locus identified in a subset analysis of chronic inflammatory diseases that included both CD and UC, and F5 and IL18R1 were within IBD risk loci.

Additionally, a number of the genes were present in GWAS loci linked to various lipid compounds and body fat metrics, such as cholesterol levels (CUX1, PXK, ITGA1, HPR, HP, and HGF), triglycerides (HPR and HGF), fatty acids (PAXIP1-DT and HPR), hormone levels (STX16 for testosterone; CPEB4, CUX1, and PXK for sex hormone-binding globulin), and weight measures including body mass index and waist-to-hip ratio (CPEB4, CUX1, GBA, GPR141, MAPK14, PXK, RPGRIP1L).

Moreover, several genes that correlated to endoscopic disease severity resided in GWAS loci linked to cell counts, including erythrocytes (CPEB4, CUX1, GBAP1, HPR, ITGA1, PXK, STX16), platelets (CPEB4, CUX1, ITGA1, PHTF1, PXK), neutrophils (IL17RA, IL18R1, PHTF1), eosinophils (CPEB4, CUX1, IL17RA, IL18R1, RNASE2, SPAG16), basophils (IL18R1, PHTF1), monocytes (IL17RA, PHTF1, TLR5), lymphocytes (IL17RA, ITGA1, PHTF1), and leukocytes (IL15, IL17RA).

Genes correlated to endoscopic disease severity were also found in GWAS loci related to liver and kidney function markers, including alanine transaminase (HPR, ITGA1), albumin (CPEB4, HPR), alkaline phosphatase (ITGA1, PHTF1, STX16), aspartate aminotransferase (HPR, PXK), creatinine (CUX1, GBAP1, PXK), gamma-glutamyl transferase (HPR, ITGA1, RPL19P5), phosphate (CUX1), urea nitrogen (GBAP1), and uric acid (CPEB4). Several genes, like CUX1, HPR, IL18R1, ITGA1, and SPAG16, were located in GWAS loci associated with levels of C-reactive protein, an inflammation marker produced by IL6-stimulation in the liver.

## Discussion

In this study, we analyzed gene expression in blood samples from CD and UC patients, both with and without bowel inflammation, and compared it to non-IBD controls. DEG highlighted biological pathways that were enriched in the 4 IBD disease states and showed correlations with endoscopic disease severity. Moreover, gene expression deconvolution revealed altered proportions of blood cell types in CD.I and UC.I compared to controls.

The most prominent pathway clusters were those observed across all 4 IBD disease states, with a notable emphasis on neutrophil degranulation. Deconvolution revealed an elevated proportion of neutrophils in peripheral blood from UC.I patients, and this increase was also seen, albeit not significantly, in the other IBD sample types. Neutrophils, while integral to antimicrobial defense, can aggravate and perpetuate inflammation and tissue damage, thus contributing to IBD pathogenesis. The potential of neutrophil-derived proteins or the neutrophil-lymphocyte ratio as blood-based IBD biomarkers has been explored.^21^, ^22^, ^23^ The neutrophil surface expression of CD64 (encoded by FCGR1A) shows promise in relation to anti-tumor necrosis factor (TNF) treatment outcome.^22^ FCGR1A emerged as a significant CEG in phagocytosis-related pathways across all sample types, showing marked upregulation in CD.I and UC.I samples. Notably, numerous neutrophil-associated genes ranked among the top 25 upregulated genes of both CD.I (eg granule proteins MPO, DEFA1, ELANE, and CTSG, and adhesion molecule CEACAM8) and UC.I (eg S100A12 and the calprotectin component S100A8 of the S100 family, and adhesion molecule CD177). CD177+ neutrophils are suggested to play a protective role in IBD due to their comparatively low levels of pro-inflammatory cytokines and enhanced antibacterial activities.^24^ Pathways like “antimicrobial peptides,” “ROS and RNS production in phagocytes,” and “RHO GTPases Activate NADPH Oxidases” were upregulated in both CD.I and UC.I samples. Neutrophil extracellular traps, comprising proteins like MPO, ELANE, calprotectin (S100A8/S100A9), and CTSG, can further intensify intestinal inflammation.^25^ Previous studies have highlighted neutrophil activation in the peripheral blood of untreated CD and UC patients.^6^

Cytokines and their receptors are central to immune response regulation, with their dysregulation impacting the pathophysiology of CD and UC. An increased understanding of these molecules enhances knowledge of disease mechanisms and aids in the development of therapeutic targets and biomarkers.^26^ Current cytokine-directed biological therapies for CD or UC include inhibition of TNF, IL12/IL23, and JAK.^26^^,^^27^ Clinical trials are evaluating additional targets, such as the IL1 receptor antagonist encoded by IL1RN,^28^ and the complex between the soluble isoform of the IL6 receptor (IL6R) and IL6.^29^^,^^30^ Among the top 25 cytokine genes, there were an upregulation of the TNF receptor TNFRSF1B in CD.I patients, a downregulation of IL23R in CD.I and UC.I patients and also IL23 in UC.I, and an upregulation of IL1RN and IL6R in UC.I. Furthermore, JAK genes were upregulated, especially in UC.I (JAK2, JAK3, and JAK1) but also in CD.I (JAK2 and JAK3). Identified cytokine expression profiles indicate potential unexplored targets or combinations for IBD treatment. In CD.I samples, RETN and FLT3 genes were most notably upregulated, whereas UC.I samples strongly associated with increased IL18R1 (including IL18RAP and IL18), IL4R, IFNAR1, and CKLF. Both CD.I and UC.I samples showed elevated IL1R2 and IL17RA. Notably, IL18RAP (alongside IL18R1, IL1R1, and IL1R2) and IL23R^5^ as well as IL6R^31^ reside within IBD GWAS loci. The potential relevance of some genes in IBD has been supported by previous studies, such as RETN with increased resistin levels in CD and primary sclerosing cholangitis,^32^^,^^33^ the FLT3 receptor in a mouse model of chronic ileitis,^34^ and the therapeutic potential of IL-18 inhibition.^35^ Though IL-17A- or IL17RA-targeting therapies are used for psoriasis, they have shown limited efficacy or adverse outcomes in CD and UC.^36^, ^37^, ^38^

The intestinal epithelial barrier separates the luminal environment from the underlying tissues and the immune cells hosted by the lamina propria and is thus considered a determinant in the etiology and pathogenesis of IBD.^39^ Cytokines like TNF, interferon-gamma, IL-1 beta, IL-4, and IL-18 are associated with an increased intestinal permeability,^40^ and in our study, their receptors (and IL18) were upregulated among the top 25 cytokine DEG. Additionally, IL18R1 showed significant correlation with endoscopic disease severity, both SAES for UC and the rescaled IBD score based on SES-CD and SAES.

A gene set analysis of DEG shared by CD and UC patients, and that correlated with the rescaled, composite endoscopic IBD grading, revealed neutrophil granules, activation, and degranulation processes, thus underscoring “neutrophil degranulation” as the top pathway in all IBD sample types. However, when considering all DEG and their associations to individual endoscopic gradings (SES-CD for CD and SAES for UC), other significant genes, compartments, processes, and pathways emerged.

Gene set analysis of DEG correlated to SES-CD highlighted the ciliary transition zone. The “Cilium Assembly” pathway was enriched in CD patients with active intestinal inflammation, but not in UC. The primary cilium is central to signaling in development and homeostasis, enabling cells to respond to external stimuli,^41^ including immune cells.^42^ RPGRIP1L is involved in processes like ciliopathies, Hedgehog signaling, and autophagy.^43^ The cilia-related SPAG16 gene has been associated to rheumatoid arthritis,^44^ possibly via extracellular matrix degradation, and a subgroup of multiple sclerosis patients express SPAG16 autoantibodies that intensify symptoms in a mouse model of the disease.^45^

Gene set analysis using DEG correlated to SAES mainly identified translation but also focal adhesion, as more significant than neutrophil degranulation. According to the pathway analysis of all analyzed genes, the cluster “major pathway of rRNA processing in the nucleolus and cytosol” was downregulated in CD and UC patients with intestinal inflammation, more so in UC. The SAES-correlated gene CPEB4, exhibiting RNA binding activity, is within an IBD GWAS locus,^5^ and autoantibodies against ribosomal protein RPS18 are potential biomarkers for early-stage Parkinson's disease.^46^ In addition to rRNA processing, this cluster also included ribosomal subunits, translation initiation, elongation, termination, and ribonucleotide modification at sites such as peptidyl tRNA delivery sites. The “tRNA processing” cluster, encompassing tRNA modification, is similarly downregulated. Ribosomal proteins, beyond their role in protein translation, participate in extra-ribosomal activities, including immune responses.^47^ Transcriptional silencing of genes coding for ribosomal proteins have revealed alterations in the expression and translation of specific subsets of genes representing functional classes such as the cell cycle, metabolism, signal transduction, and cell response.^48^ In mice, macrophage-specific L13a ribosomal protein deficiency disrupts an L13a-dependent translational silencing mechanism, elevating several chemokines.^49^ Moreover, the ribosomal proteins RPL9 and RPS5 can dampen inflammatory responses, possibly by interacting with lipopolysaccharide and inhibiting downstream activation.^50^ Processing and modification of tRNA also play roles in physiological and pathological processes, including immune regulation,^51^ with, for example, a decrease in tRNA gene transcription during monocyte-to-macrophage differentiation, and altered immune cell populations in mice deficient in the tRNA methyltransferase FTSJ1. Of note, FTSJ1 was differentially expressed in UC.I patients. Taken together, these observations indicate that an altered processing of rRNA and tRNA leads to modification of protein synthesis and intracellular trafficking with bearing on central immune regulatory functions of relevance to IBD, albeit further characterization of IBD pathogenesis is needed.

Utilizing the GWAS Catalog, 5 genes associated with endoscopic disease severity (namely CPEB4, F5, GBAP1, IL18R1, and PHTF1) were located in IBD GWAS risk loci. The gene GBA, which correlated to the rescaled IBD score and is situated close to the pseudogene GBAP1, is noted as being linked to a GWAS significant risk locus for CD.^5^ Moreover, additional genes identified in relation to endoscopic disease severity were connected to genes present in IBD GWAS risk loci. IL15RA, located in an IBD risk locus,^52^ encode a cytokine receptor for IL15, which was identified in relation to the SES-CD scale. STX16 which correlated to the SAES scale is involved in SLC2A4 trafficking,^53^ and the SLC2A4-regulator SLC2A4RG is in an IBD-associated risk locus.^5^ HGF was identified in relation to the rescaled IBD score, and the HGF activator HGFAC is located within an IBD risk locus.^5^ The pseudogene RPS2P14 (rescaled IBD score) is in an intron of PHTF1 (SAES score). Pseudogenes and long intergenic nonprotein coding RNAs may regulate mRNA silencing through microRNAs.^54^ Apart from pseudogenes in IBD risk loci, we identified long intergenic nonprotein coding RNAs (eg LINC02981) and microRNAs (eg MIR7848). In IBD, noncoding RNAs relate to immune responses and the intestinal barrier.^55^^,^^56^

In total, twelve of the genes that correlated with endoscopic disease severity were associated to various lipid compounds and body fat metrics. Additionally, TANC2 has been identified in a cholesterol homeostasis-related module of Alzheimer’s disease DEG.^57^ Studies indicate a connection between the levels of different bioactive fatty acids and lipid compounds with IBD, influencing inflammatory responses and impacting the severity of the disease.^58^, ^59^, ^60^ Similarly, there is evidence suggesting that body composition factors, such as obesity, can influence disease activity, extraintestinal manifestations, and response to treatment.^61^

Furthermore, fourteen of the genes that correlated to endoscopic disease severity resided in GWAS loci were linked to cell counts, including erythrocytes, neutrophils, eosinophils, basophils, monocytes, lymphocytes, and leukocytes, suggesting potential relevance of these cell types to IBD severity. The associations between gene expression in blood and endoscopic disease severity might arise from covariation of disease-related cell types, cell dysfunction, or pleiotropic effects of the genes. Various blood cell count measures have been suggested in the assessment of IBD, for example, neutrophil-to-lymphocyte ratio and platelet-to-lymphocyte ratio,^62^ platelet-to-albumin ratio and the percent volume of platelets in the blood ,^63^ mean platelet volume,^64^ and red cell distribution width.^65^ It has further been proposed that neutrophil activation reflects the disease activity in CD and UC, whereas the pattern of eosinophil activation depended on the disease subtype.^66^

A number of genes correlated to endoscopic disease severity were involved in tissue repair and epithelial barrier dysfunction, thus underlining a role in IBD pathophysiology.^67^ The ZNF582-encoded protein binds to and increase the expression of TJP2,^68^ a tight junction protein linked to a genetic cause of cholestasis.^69^ The noncoding PAXIP1-DT promotes cell proliferation, migration, apoptosis, and possibly epithelial-mesenchymal transition.^70^ STX16 depletion compromises barrier function, impairs E-cadherin recycling, and disrupts epithelial lumen formation in vitro.^71^ The transcription factor CUX1 regulates genes involved in cytoskeleton function and cell motility, and play a role intestinal epithelial wound healing and barrier maintenance.^72^ Additionally, CUX1 interacts with the protective promoter allele of the JAK2 gene, present in an IBD risk locus.^73^ In a CD-like ileitis mouse model, TLR5 activation increased epithelial permeability and reduced tight junction protein expression.^74^ Pertinent to the primary cilia proteins RPGRIP1L and SPAG16, gene silencing related to ciliogenesis increased the expression of markers associated with epithelial-mesenchymal transition.^75^

Hepatobiliary and renal involvement are extraintestinal manifestations of IBD.^76^^,^^77^ Nine of the genes that correlated to endoscopic disease severity were found in GWAS loci related to liver and kidney function markers, including alanine transaminase, albumin, alkaline phosphatase, aspartate aminotransferase, creatinine, gamma-glutamyl transferase, phosphate, urea nitrogen, and uric acid. Three of these 9 genes and an additional 2 genes were located in GWAS loci associated with levels of C-reactive protein, an inflammation marker produced by IL6-stimulation in the liver. In an evaluation of blood parameters, including, for example, a complete blood count, albumin, C-reactive protein, and IL6, both high sensitivity C-reactive protein and C-reactive protein-to-albumin ratio related to the endoscopic activity of UC.^78^ Moreover, haptoglobin (HP), an indicator of liver function and a scavenger of free hemoglobin in plasma, was among the genes correlated to endoscopic disease severity. Free hemoglobin alpha is detected in inflamed colonic tissues of both CD and UC patients,^79^ and therapeutic hemoglobin and hemin scavengers, such as haptoglobin and hemopexin, have been discussed in pathologies initiated by extracellular hemoglobin.^80^

Some of the genes correlated to endoscopic disease severity showed relevance in relation to contemporary IBD treatments, drug targets, or drug repurposing opportunities. In CD patients, responders to infliximab, compared to nonresponders, present elevated pretreatment serum IL15 levels and decreased post-treatment levels.^81^ A genetic variant of GPR141 is linked to an accelerated onset of herpes zoster in patients with rheumatoid arthritis and psoriasis undergoing treatment with the JAK inhibitor tofacitinib,^82^ a JAK inhibitor also used in the treatment of UC.^83^ In a mouse model of UC, emodin reduced symptoms, inflammation, intestinal damage, expression of TLR5, as well as serum levels of antibodies directed against the TLR5 ligand bacterial flagellin.^84^ The pseudogene GBAP1 is GWAS associated with drugs targeting the renin-angiotensin system,^85^ a system that has been suggested as a novel target in IBD management.^86^ Riluzole, an approved drug for amyotrophic lateral sclerosis that inhibits glutamate release and augments the glutamate transporter SLC1A3,^87^ has been suggested for repositioning to an anti-colitic drug.^88^ Given that several genes associated with endoscopic disease severity were linked to traits such as levels of cholesterol, triglycerides, and fatty acids, statins might prove beneficial in IBD treatment. However, their efficacy remains to be determined.^89^

## Conclusion

In order to devise more efficient treatments, it is imperative to gain a deeper understanding of the intricate factors contributing to IBD. This study revealed biological pathways associated with IBD disease state and endoscopic disease severity, thereby providing insights into the underlying mechanisms of IBD pathogenesis, as well as identifying potential biomarkers and therapeutic targets for disease management. Besides supporting current knowledge of IBD mechanisms, our study suggests additional pathways, particularly concerning neutrophil degranulation and cytokine networks, as well as less investigated biomarkers and drug targets that deserve further consideration. Additionally, we have explored associations with other measures such as cholesterol levels, blood cell counts, and markers assessing liver and kidney function. The predictive value of these genes in relation to, for example, endoscopic active disease needs to be investigated further in a larger patient cohort.

Peripheral blood might constitute a suitable noninvasive diagnostic sample type, with gene expression profiles as indicators of mucosal inflammation and engaged pathways that guide personalized treatment decisions. Further research is, however, warranted to validate and refine these findings.

## Supplementary Materials

Table A1 Proportions of peripheral blood cell types for each study subject, estimated using gene expression deconvolution.

Table A2 Genes differentially expressed in peripheral blood from study subjects with an inflamed intestinal mucosa (CD.I and UC.I) or with a non-inflamed intestinal mucosa (CD.nI and UC.nI) compared to peripheral blood from controls (Cntrl).

Table A3 Gene set enrichment analysis of peripheral blood from CD or UC study subjects, with or without an inflamed intestinal mucosa, compared to peripheral blood from controls.

Table A4 Core enrichment and/or differentially expressed cytokine and cytokine receptor genes for each of the 4 sample type comparisons, that is, peripheral blood from CD or UC study subjects, with or without an inflamed intestinal mucosa, compared to peripheral blood from controls.

Table A5 Correlations between endoscopic activity scores and gene expression in peripheral blood samples. The table contains the following correlations: 1) the simplified endoscopic activity scores for CD patients (SES-CD) vs CD DEG, 2) the sum of the segmental assessment of endoscopic severity for UC patients (SAES) vs UC DEG, 3) UC endoscopic index of severity (UCEIS) vs UC DEG, 4) the rescaled IBD score based on the SES-CD and SAES vs DEG in common between CD and UC patients, and 5) the rescaled IBD score based on the SES-CD and UCEIS vs DEG shared between CD and UC patients.

Table A6 Over-representation analysis (ORA) of genes whose expression correlated with endoscopic activity score. ORA was performed on the following data sets: 1) genes whose expression correlated, at a nominal *P* < .05, to the simplified endoscopic activity score (SES-CD) for CD patients, 2) genes whose expression correlated, after adjustment for multiple testing, to the sum of the segmental assessment of endoscopic severity (SAES) for UC patients, and 3) DEG shared between CD and UC patients whose expression correlated, after adjustment for multiple testing, to the rescaled IBD score that was based on the SES-CD scale for CD patients and the SAES scale for UC patients.
